# Bifunctional CePO_4_/CeO_2_ nanocomposite as a promising heterogeneous catalyst for the enhancement of the ozonation recovery effect in the presence of chloride ions

**DOI:** 10.1038/s41598-022-13069-5

**Published:** 2022-05-31

**Authors:** Lilla Fijołek, Lukasz Wolski

**Affiliations:** Faculty of Chemistry, Adam Mickiewicz University, Poznań, Uniwersytetu Poznańskiego 8, 61-614 Poznań, Poland

**Keywords:** Environmental chemistry, Catalysis

## Abstract

The degradation of organics through ozonation is strongly reduced by chloride ions. Although the efficiency of such processes can be recovered in the presence of homogeneous phosphates, the addition of these chemicals to water is problematic because of the generation of secondary wastes. Phosphates are known as one of the most important biogens responsible for the eutrophication of rivers and lakes. Thus, their worldwide application should be limited. The main goal of this work was to characterize the performance of solid-state cerium(III) phosphate (CePO_4_), cerium dioxide (CeO_2_), and bifunctional CePO_4_/CeO_2_ nanocomposite as substitutes for homogeneous phosphates during the ozonation of benzoic acid (BA) in the presence of chlorides. All solid-state samples used in this study were synthesized by facile hydrothermal method and thoroughly characterized. It was documented that heterogeneous CePO_4_ showed significantly better ozonation recovery effect than homogeneous phosphates. It was also established that the process efficiency could be further enhanced by using the bifunctional nanocomposite. Tests with the use of *tert*-butanol as a hydroxyl radical scavenger revealed that the improved ozonation efficiency in the presence of CePO_4_/CeO_2_ resulted from the action of HO^•^ radicals which were the key reactive oxygen species responsible for the recovery of BA degradation in the presence of chlorides.

## Introduction

For the last few decades, catalytic ozonation has been attracting particular attention as one of the most promising methods for elimination of organic pollutants from contaminated water^1^. Numerous studies have revealed, however, that the efficiency of ozonation processes is strongly lowered by the presence of chlorides or bromides^2^. According to the previous reports^3^, the inhibiting effect of chlorides can be eliminated by addition of phosphate ions to the solution. Although phosphates can overcome the scavenging effect of chlorides, their use is problematic because they significantly contribute to eutrophication of natural waters^4^. For this reason, development of new heterogeneous catalysts that would be easier to separate from the reaction mixture after the ozonation process, has attracted particular attention in terms of water and wastewater purification.

Until now, many authors have reported that the efficiency of ozonation processes can be enhanced in the presence of heterogeneous catalysts^5–8^. More efficient degradation of organic pollutants through catalytic ozonation is possible because of the unique ability of a selected nanomaterial (e.g. MnO_2_, Fe_2_O_3_, FeOOH, NiO, Co_3_O_4_ or CeO_2_) to catalyze ozone decomposition toward the formation of other strongly oxidizing reactive oxygen species (ROS) (e.g., hydroxyl radicals, singlet oxygen, etc.)^1,9–11^. Concerning ozonation processes in the presence of CeO_2_ as catalyst, it was found that reactivity of this metal oxide is strongly affected by the size of the ceria particles and concentration of lattice defects (i.e., Ce^3 +^ ions and oxygen vacancies)^8^. According to recent studies by Wang et al.^12^, Ce^3+^ ions are one of the key species responsible for the catalytic activation of ozone on CeO_2_ towards formation of hydroxyl radicals via reactions (1–2).

1 $${\text{Ce}}^{{{3} + }} + {\text{ O}}_{{3}} + {\text{ H}}^{ + } \to {\text{ Ce}}^{{{4} + }} + {\text{ HO}}_{{3}}^{ \bullet }$$

2 $${\text{HO}}_{{3}}^{ \bullet } \to {\text{ HO}}^{ \bullet } + {\text{ O}}_{{2}}$$

To the best of our knowledge, there are no literature reports on the catalytic ozonation in the presence of heterogeneous metal phosphates. Regarding the other advanced oxidation processes, cerium(III) phosphate (CePO_4_) has been documented to be a promising catalyst for activation of hydrogen peroxide (H_2_O_2_) to form highly oxidizing ROS^13^. Interestingly, the highest peroxidase activity was not observed for the pure CePO_4_ catalyst, but for a mixture of CeO_2_ and CePO_4_. The superior activity of this mixed catalytic system originated from enhanced redox switching between Ce^3+^  ↔ Ce^4+^ sites from the CePO_4_ and CeO_2_ lattice, respectively. One can expect that this unique feature of mixed CePO_4_-CeO_2_ catalysts should also play important role in catalytic activation of ozone, while the presence of inorganic phosphates in the catalysts, should eliminate the inhibiting effect of chlorides during ozonation process, leading to more efficient degradation of organic pollutants than that observed for sole CeO_2_ or CePO_4_ as well as homogeneous phosphate ions.

The main goal of this work was to investigate the impact of solid-state cerium(III) phosphate (CePO_4_), cerium dioxide (CeO_2_), and bifunctional CePO_4_/CeO_2_ nanocomposite on the recovery efficiency of ozonation processes in the presence of chloride anions. The studies included preparation, characterization, and evaluation of the catalytic activity of the materials in the degradation of benzoic acid as a model organic pollutant. The activity of the materials synthesized in this work was compared with those of homogeneous systems in which the phosphate anions recovered the self-enhanced ozonation of BA known from the literature^3^.

## Results and discussion

### Characterization of catalysts

**Figure 1:**
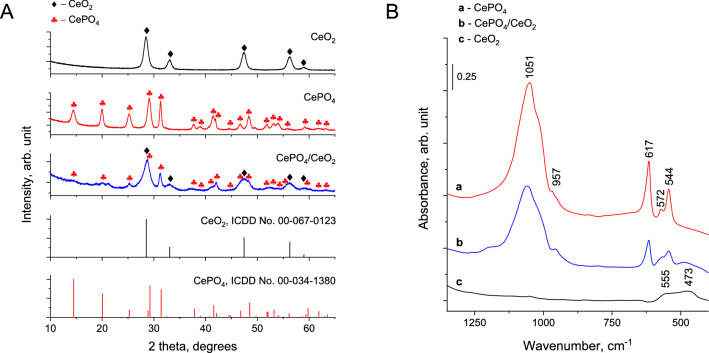
(**A**) XRD patterns and (**B**) FTIR spectra of the catalysts.

Figure 1A shows the XRD patterns of the catalysts prepared in this study. Cerium dioxide was found to crystallize in cubic CeO_2_ phase (ICDD entry number: 00-067-0123), while cerium(III) phosphate had a hexagonal CePO_4_ structure (ICDD entry number: 00-034-1380). For the bifunctional CePO_4_/CeO_2_ nanocomposite, one can observe the XRD peaks typical of both CeO_2_ and CePO_4_ phases. Interestingly, the characteristic reflections of CeO_2_ and CePO_4_ in the bifunctional sample were significantly less intense than those observed for the sole CeO_2_ and CePO_4_, indicating a lower crystallinity or smaller crystallite size of these phases in the nanocomposite.

**Figure 2:**
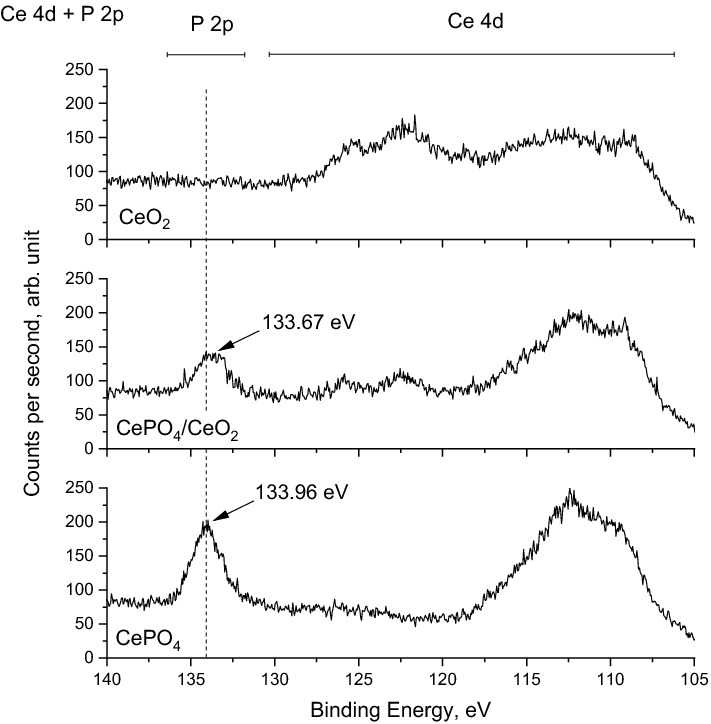
Ce 4d and P 2p high resolution XPS spectra of the catalysts prepared in this work.

The successful formation of the bifunctional CePO_4_/CeO_2_ catalyst was also indicated by FTIR spectroscopy. As depicted in Fig. 1B, the IR spectrum of the parent CeO_2_ revealed the presence of broad absorption bands at ca. 555 and 473 cm^‒1^, which are characteristic of Ce‒O stretching vibrations in the structure of CeO_2_^14^. The vibrational bands mentioned above were not identified for the parent CePO_4_ for which the most intense IR bands were found at ca. 1051, 617 and 544 cm^‒1^. According to the literature^15^, these IR bands are typical of P‒O stretching, O=P‒O bending, and O‒P‒O bending vibrational modes of PO_4_^3‒^ groups in CePO_4_. The IR spectrum of the bifunctional sample shows all the above-mentioned vibrational bands typical of both CeO_2_ and CePO_4_. Therefore, similar to the XRD results, FTIR analyzes indicated that the bifunctional catalyst consisted of two different phases, namely CeO_2_ and CePO_4_. The presence of phosphates on the surface of CePO_4_ and CePO_4_/CeO_2_ was further confirmed by X-ray photoelectron spectroscopy. As shown in Fig. 2, only for these two samples, the P 2p peak, typical of phosphate ions, was identified^16^. Interestingly, the P 2p peak in the spectra of the bifunctional catalyst was characterized by the slightly lower binding energy than that in the parent CePO_4_ (133.67 eV vs. 133.96 eV, respectively; Fig. 2). This slight shift of the P 2p peak may indicate on the presence of a strong electronic interaction between CePO_4_ and CeO_2_ species in the bifunctional nanocomposite.

**Figure 3:**
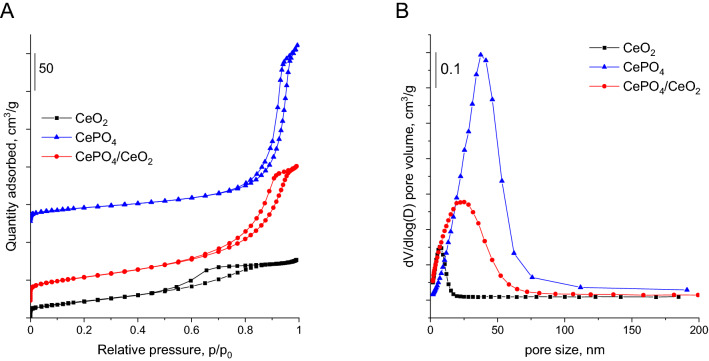
(**A**) Nitrogen adsorption–desorption isotherms recorded for the catalysts. (**B**) Pore size distribution estimated for the catalysts from the adsorption branches of isotherms using BJH method.

The porosities of the catalysts were compared on the basis of nitrogen physisorption measurements (Fig. 3A). It was found that all materials had porous structure but exhibited a different pore size distribution (PSD). The smallest and the most homogeneous mesopores (approximately 7 nm in diameter) were observed for CeO_2_ (Fig. 3B). In the case of CePO_4_, the pores were significantly larger and PSD was much broader. As depicted in Fig. 3B, this material contained mainly large mesopores with the size of ca. 40 nm and some macropores with a size greater than 50 nm. In contrast, CePO_4_/CeO_2_ contained both small mesopores characteristic of CeO_2_ and larger mesopores and macropores typical of CePO_4_ (Fig. 3B). Thus, nitrogen physisorption measurements revealed that the bifunctional catalyst consisted of two different phases fused into a porous structure. Nitrogen physisorption measurements allowed also for the estimation of BET surface area of the catalysts. It was found that the greatest surface area was characteristic of the bifunctional CePO_4_/CeO_2_ (119 m^2^/g). For the parent CeO_2_ and CePO_4_ samples, the surface areas were slightly smaller (78 and 73 m^2^/g, respectively).

**Figure 4:**
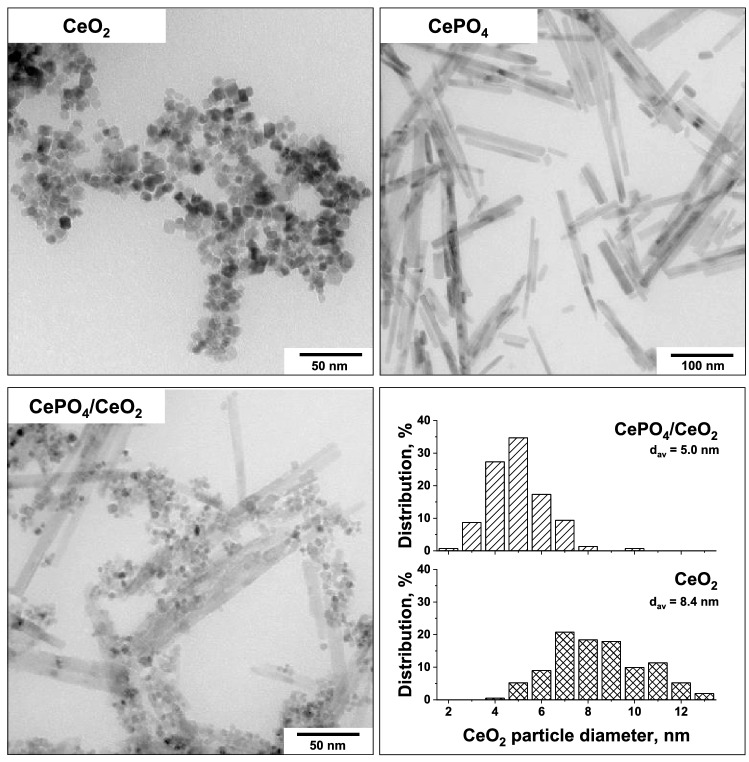
TEM images of the catalysts and ceria particle size distribution estimated for CeO_2_ and CePO_4_/CeO_2_.

The morphology of the catalysts was characterized with the use of transmission electron microscopy (Fig. 4). It was found that the parent CeO_2_ consisted of small polyhedral particles. In contrast to CeO_2_, the CePO_4_ particles had a rod-like shape with a diameter of ca. 10–15 nm, and length from ca. 50 nm up to 300 nm. Results obtained from TEM measurements further confirmed that the bifunctional CePO_4_/CeO_2_ catalyst is a nanocomposite consisting of small polyhedral particles of CeO_2_ supported on rod-like particles of CePO_4_. As shown in Fig. 4, the CeO_2_ particles in the bifunctional catalyst were smaller than those observed for the parent CeO_2_. In view of these results, one can conclude that the strong interaction between the CePO_4_ and CeO_2_ phases in the bifunctional sample (indicated by XPS) resulted in a better stabilization of ceria particles on the surface of CePO_4_, and this inhibited their agglomeration during the hydrothermal synthesis.

### Catalytic ozonation recovery effect in the presence of chlorides

**Figure 5:**
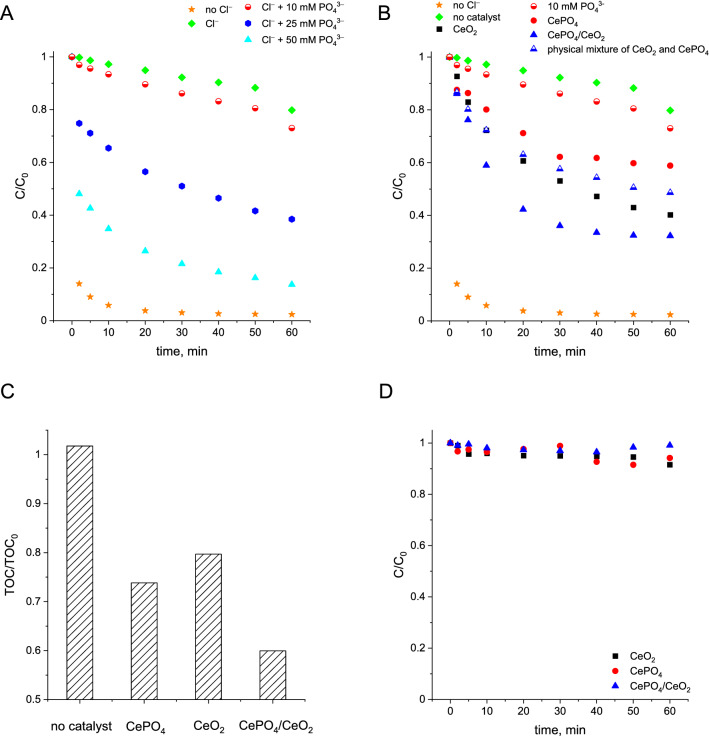
(**A**) Influence of phosphate concentration on the efficiency of benzoic acid degradation during ozonation in the presence of chloride ions; (**B**) Results of benzoic acid degradation during ozonation processes in the presence of chlorides and heterogeneous catalysts synthesized in this study; (**C**) Total organic carbon removal after 60 min of ozonation processes in the presence of chlorides and catalysts. (**D**) Removal of benzoic acid from reaction mixtures resulting from adsorption of BA on the catalyst surfaces in the absence of ozone; *Reaction conditions:* 200 mL of BA solution (24 µM), initial ozone concentration: 120 µM, initial pH: 2.5, concentration of chloride ions: 3.22 mM, catalyst loading: 0.2 g/L, room temperature. The physical mixture of CeO_2_ and CePO_4_ consisted of 20 mg of cerium dioxide and 20 mg of cerium(III) phosphate.

The degradation efficiency of benzoic acid through ozonation in the presence or absence of chloride ions is shown in Fig. 5A. Similar to previous studies^3^, we have found that BA is completely degraded by ozone after 10 min of the reaction, but addition of chloride ions to the reaction mixture significantly suppresses the efficiency of the degradation process. In the presence of 3.22 mM chlorides, degradation of benzoic acid by ozonation process was almost completely inhibited (Fig. 5A). The efficiency of BA degradation in the presence of chloride ions is recovered by the addition of homogeneous phosphates but this effect is clearly noticeable only at relatively high concentrations of phosphate ions (up to 50 mM; Fig. 5A).

Figure 5B shows the influence of the heterogeneous catalysts synthesized in this study on the efficiency of BA degradation by ozone in the presence of chloride ions. It was found that the addition of heterogeneous cerium phosphate enabled for a more efficient ozonation recovery than that previously observed for homogenous process. The positive impact of CePO_4_ was noticeable even at a very low catalyst loading of 0.2 g/L. It means that CePO_4_ containing approximately 1 mM of PO_4_^3‒^ in a solid form exhibits significantly better ozonation recovery performance than that of a much greater amount of homogeneous phosphates (i.e. 10 mM see Fig. 5B). More pronounced increase in efficiency of BA degradation in the presence of chloride ions was observed for the parent CeO_2_. This material was ca. 2 times more active than CePO_4_. Interestingly, the highest BA degradation was found for the bifunctional nanocomposite. This material was not only more active than the sole CePO_4_ or CeO_2_, but also the physical mixture of CeO_2_ and CePO_4_ (Fig. 5B). The bifunctional nanocomposite catalyst also exhibited the highest ability to mineralize the organic pollutant (Fig. 5C). To confirm that the enhanced reactivity of CePO_4_/CeO_2_ did not result from the adsorption of benzoic acid on its surface, additional adsorption experiments were performed. As depicted in Fig. 5D, all catalysts used in this study exhibited a negligible ability to adsorb BA. In view of all these results, one can clearly conclude that the increased reactivity of CePO_4_/CeO_2_ must originate from the synergistic interaction between CePO_4_ and CeO_2_ in the bifunctional nanocomposite.

**Figure 6:**
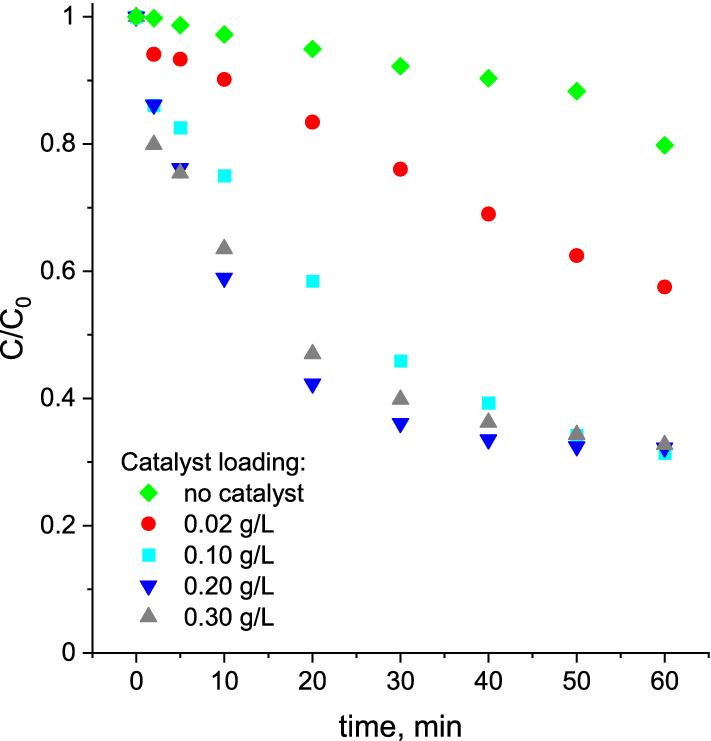
The influence of CePO_4_/CeO_2_ dosage on the efficiency of ozonation recovery in the presence of chloride ions. *Reaction conditions:* 200 mL of BA solution (24 µM), initial ozone concentration: 120 µM, initial pH: 2.5, concentration of chloride ions: 3.22 mM, room temperature.

In order to investigate the influence of catalyst loading on the efficiency of ozonation recovery, additional catalytic tests with the use of various amounts of the catalyst were performed. As shown in Fig. 6, the positive impact of the CePO_4_/CeO_2_ catalyst on the ozonation recovery in the presence of chloride ions was clearly noticeable at a very low catalyst loading of 0.02 g/L. Such a low catalyst loading enabled for a more efficient ozonation recovery than a much greater amount of homogeneous phosphates (compare Figs. 5A and 6). When the catalyst concentration increased from 0.02 g/L to 0.10 g/L, the conversion of BA increased from about 42.5 to 67.7%. Further increase of the catalyst loading did not result in a significant enhancement of the ozonation recovery effect but allowed higher BA degradation rate at the beginning of the ozonation process (Fig. 6). The optimal catalyst dosage for the most efficient ozonation recovery in a short reaction time was established at 0.20 g/L.

**Figure 7:**
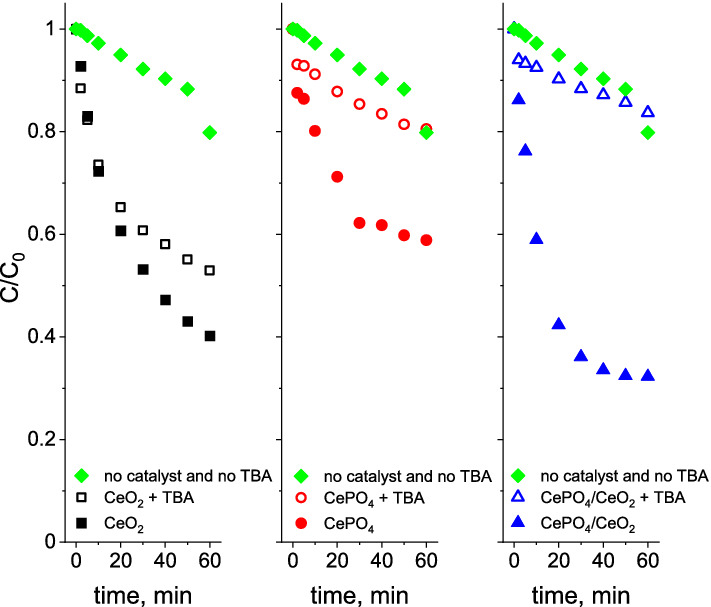
Influence of hydroxyl radical scavenger (*tert*-butyl alcohol) on the efficiency of ozonation processes in the presence of chloride ions. *Reaction conditions:* 200 mL of BA solution (24 µM), initial ozone concentration: 120 µM, initial pH: 2.5, concentration of chloride ions: 3.22 mM, concentration of *tert*-butyl alcohol: 4 mM, catalyst loading: 0.2 g/L, room temperature.

To shed more light on the origin of the recovery of ozonation efficiency in the presence of the bifunctional CePO_4_/CeO_2_ catalyst, additional tests with the use of *tert*-butyl alcohol (TBA) as the hydroxyl radical scavenger were performed. As shown in Fig. 7, the presence of TBA in the solution totally inhibits the ozonation recovery effect observed both for the sole CePO_4_ and the bifunctional CePO_4_/CeO_2_ catalyst. This observation allowed for conclusion that the ozonation recovery effect in the presence of these two samples resulted from the efficient action of strongly oxidizing hydroxyl radicals. Interestingly, a different effect of the TBA scavenger was observed for the reaction with the use of CeO_2_ as a catalyst, in which TBA had only a slight impact on the ozonation process (Fig. 7). It showed that the mechanism of the catalytic recovery process in the presence of sole CeO_2_ must differ from that observed for samples containing cerium(III) phosphate (i.e. CePO_4_ and CePO_4_/CeO_2_), and results more likely from formation of other reactive oxygen species characterized by non-radical character. According to previous literature reports^17–20^, ozone activation over transition metal oxides may result not only in the formation of hydroxyl radicals but also singlet oxygen and other surface adsorbed activated oxygen species (e.g. surface atomic oxygen (*O) or surface peroxide species (O_2_^2‒^)). The as-formed nonradical ROS are also capable to oxidize various organic compounds^18–20^. Concerning other non-radical mechanisms of organics degradation by ozone, it has been documented that organic molecules can be destroyed via the intramolecular electron transfer process from adsorbed O_3_ by the formation of bridging or complexing structures^18,20^. A similar reaction mechanism has previously been reported for degradation processes with the use of hydrogen peroxide as an oxidant, in which the surface peroxide-like species formed on the surface of CeO_2_ were found to be responsible for the efficient degradation of various organic dyes^21–23^. The possible mechanism of BA degradation over CeO_2_ via the nonradical pathway will be further discussed in Sect. “Discussion”.

The mentioned above nonradical mechanism of ozone activation in the presence of sole CeO_2_ indicates that the main component of the bifunctional CePO_4_/CeO_2_ catalyst responsible for the improved ozonation recovery effect is CePO_4_. However, it is important to emphasize that CePO_4_ used alone was not as active as the bifunctional catalyst (Fig. 5B). Therefore, the presence of strong interface interactions between CePO_4_ and CeO_2_, allowing for the synergistic effect of these two components of the bifunctional nanocomposite, was essential to obtain the improved recovery effect during BA ozonation.

### Discussion

The degradation of BA by ozone is known as a self-enhanced process that is initiated by a series of slow reactions between ozone and BA, resulting in the formation of hydroxyl radicals^24^. The as-formed hydroxyl radicals react then with BA leading to its efficient oxidation (the rate of reaction (k) between BA and hydroxyl radicals is equal to 5.5 × 10^9^ M^−1^ s^−1^)^25^. However, the efficiency of the self-enhanced ozonation process is greatly reduced in the presence of chloride ions that act as a hydroxyl radicals scavenger^3,26^. As indicated in this study, in the presence of 3.22 mM chlorides, degradation of benzoic acid by ozone was almost completely inhibited (Fig. 5A). The scavenging effect of chlorides results from the fact that they can react with hydroxyl radicals via reaction (3) leading to formation of HOCl^•‒^ (reaction rate = 4.03 ± 0.4 × 10^9^ M^‒1^ s^‒1^)^26^. This reaction is reversible and HOCl^•‒^ species can dissociate back to hydroxyl radicals and chloride ions (k = 6.1 ± 0.8 × 10^9^ s^‒1^)^26^. However, at low pH values, HOCl^•‒^ species can also be converted to Cl^•^ radicals by reaction (4) (k = 2.1 ± 0.7 × 10^10^ M^‒1^ s^‒1^)^26^. The lower the pH, the more efficient transformation of HOCl^•−^ into Cl^•^ . At pH below 7.2 Cl^•^ are the dominant species^26^.

3 $${\text{HO}}^{ \bullet } + {\text{ Cl}}^{ - } \leftrightarrow {\text{ HOCl}}^{ \bullet - }$$

4 $${\text{HOCl}}^{ \bullet - } + {\text{H}}^{ + } \leftrightarrow {\text{ Cl}}^{ \bullet } + {\text{ H}}_{{2}} {\text{O}}$$

In view of the above information, one can conclude that the rate of the reaction between hydroxyl radicals and chloride ions is only slightly lower than that established for the reaction between hydroxyl radials and BA (k = 4.03 ± 0.4 × 10^9^ M^‒1^ s^‒1^^26^ vs. k = 5.5 × 10^9^ M^‒1^ s^‒1^^25^, respectively). Therefore, when the concentration of chloride ions is significantly higher than the concentration of BA, the chloride ions can totally quench the degradation of BA by hydroxyl radicals (Fig. 5A). According to the literature^26,27^, the quenching effect of chloride ions is affected by the pH of the reaction mixture. The lower the pH of the reaction mixture, the stronger the scavenging effect, what is caused by the more efficient transformation of HOCl^•‒^ into Cl^•^ via reaction (4)^26,27^. It means that at low pH values and high concentration of chloride ions, hydroxyl radicals formed by the reactions between BA and ozone are immediately transformed by reactions (3) and (4) into significantly less reactive Cl^•^ radicals. Furthermore, one cannot exclude that the as-formed radicals may then react with chlorides via reaction (5), leading to the formation of Cl_2_^•^‾ (k = 8.5 × 10^9^ M^−1^ s^−1^^28,29^, or k = 2.1 × 10^10^ M^−1^ s^−1^^30^).

5 $${\text{Cl}}^{ \bullet } + {\text{ Cl}}^{ - } \to {\text{ Cl}}_{{2}}^{ \bullet - }$$

The radical Cl_2_^•^‾, which is predominantly formed at low pH^31^, cannot oxidize benzoic acid^32^. Since both Cl^•^ and Cl_2_^•^ radials are not able to degrade BA, the degradation of BA is totally quenched in the presence of a large excess of chloride ions.

The influence of phosphate ions on the ozone decomposition processes has not yet been clarified. Some researchers indicated that phosphate ions inhibit ozone decomposition^33–36^, while others revealed that phosphate species accelerate ozone decomposition^34,37^. The latter effect was found to be the most pronounced at low pH^38^. In 1985, Staehelln and Hoigne^34^ established that phosphate ions do not react with ozone; however, they can react with HO^•^ radicals. According to these authors, phosphate radicals formed in this reaction are capable of abstracting a hydrogen atom from some types of organic compounds^34^. The ability of phosphate radicals to oxidize organics was confirmed by Rosso et al.^39^. Thus, phosphate ions can interfere not only with radical-type chain reactions during ozonation processes, but also act as a secondary oxidant facilitating the organics degradation.

Previous reports have shown that phosphates can enhance the efficiency of the ozonation process in the presence of chlorides^3^. However, the role of phosphate ions in recovery of the ozonation process has not been clarified yet. As depicted in Fig. 5A, the recovery of self-enhanced ozonation of BA in the presence of chlorides is observed only at a relatively high excess of homogeneous phosphate ions. In view of these observations, one can expect that phosphate radicals, which are capable to oxidize benzoic acid, may hinder the scavenging effect of chloride ions but only when the former are formed in a greater amount than the less reactive Cl^•^ radicals. The efficiency of the ozonation process cannot be fully recovered due to probable formation of some Cl^•^ radicals via reaction (6) (k = 2.2 × 10^6^ M^−1^ s^−1^)^40^ that results in lowering of the positive effect of phosphates on the recovery of ozonation.

6 $${\text{H}}_{{2}} {\text{PO}}_{{4}}^{ \bullet } + {\text{ Cl }}^{ - } \to {\text{ H}}_{{2}} {\text{PO}}_{{4}}^{ - } + {\text{ Cl}}^{ \bullet }$$

As concerns catalytic ozonation, the strongly enhanced reactivity of the heterogeneous cerium phosphate results from the fact that this heterogeneous catalyst plays two different roles at the same time. Firstly, Ce^3+^ ions from CePO_4_ promote more efficient activation of ozone towards formation of hydroxyl radicals via reaction (1) and (2)^12^. The more efficient formation of hydroxyl radicals through the activation of ozone by CePO_4_, the higher the probability of reaction between ROS and BA, and thus the less pronounced reaction quenching effect caused by chloride ions.

Further, ozone activation over CePO_4_ take place on the surface of this heterogenous catalyst. Thus, one cannot completely exclude the possibility that surface phosphate species may react with the as-formed hydroxyl radicals leading to the generation of surface phosphate radicals. The phosphate radicals may then react with BA resulting in its degradation. Since the ozonation recovery effect in the presence of CePO_4_ is inhibited by addition of TBA, which also can scavenge phosphate radicals (Fig. 7), the hypothesis about formation of surface phosphate radicals is very probable.

**Figure 8:**
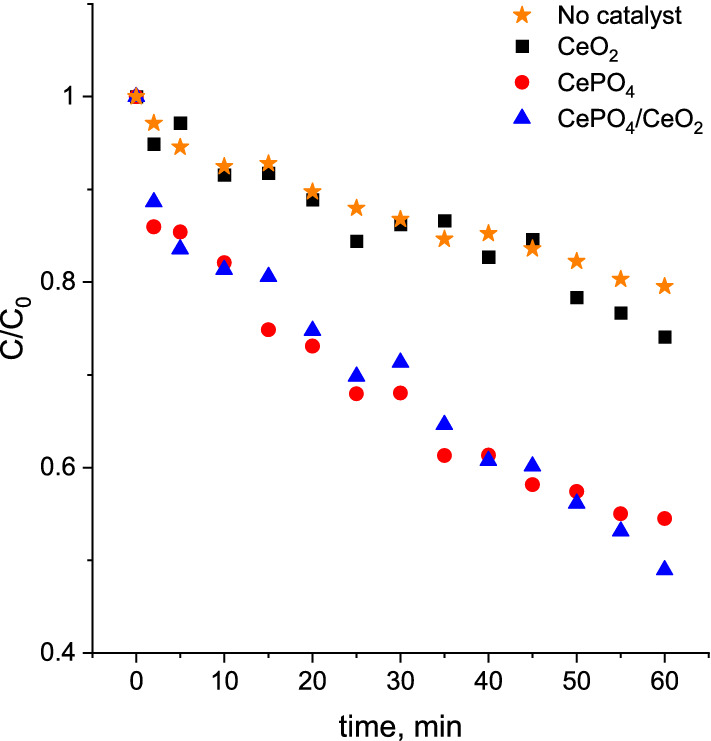
Efficiency of ozone decomposition in the presence of the catalysts. *Reaction conditions:* initial ozone concentration: 120 µM, initial pH: 2.5, catalyst loading: 0.2 g/L, room temperature. Reactions conducted without BA and chloride ions in the solution.

Tests with the use of a TBA indicated that BA degradation over CeO_2_ proceeds mainly by the nonradical pathway (Fig. 7). On the contrary, the degradation of BA over the bifunctional CeO_2_/CePO_4_ nanocomposite proceeded according to the same radical pathway as that observed for the CePO_4_. The different reactivity of CeO_2_ was also observed in ozone decomposition tests. As shown in Fig. 8, CeO_2_ exhibited no activity in ozone decomposition, while all phosphate-containing catalysts were more active in this process. Since ozone cannot be efficiently activated on ceria towards formation of hydroxyl radicals, we claimed that the main ROS responsible for degradation of BA in the presence of CeO_2_ could be an surface atomic oxygen and/or surface peroxide species (O_2_^2‒^). This hypothesis is supported by previous literature reports^41^ which indicated that these two above-mentioned nonradical ROS are one of the key active species responsible for efficient degradation of p-nitrophenol during the catalytic ozonation in the presence of CeO_2_. Furthermore, the above results of ozone decomposition clearly indicate that the main active component of the nanocomposite responsible for activation of ozone and degradation of BA via the radical pathway is CePO_4_. The strongly enhanced reactivity of the bifunctional nanocomposite results most probably from the presence of a strong interface between CeO_2_ and CePO_4_. According to the previous reports^13^, such an interface may facilitate redox switching between Ce^3+^  ↔ Ce^4+^ sites from the CePO_4_ and CeO_2_ lattice, respectively. Vinothkumar et al.^13^ have shown that this enhanced redox switching promotes more efficient activation of H_2_O_2_ toward the formation of hydroxyl radicals. We hypothesize that the same synergistic effect is also observed in this study during ozone activation. This hypothesis is in agreement with the experimental data which indicated that the synergistic effect is not observed for the physical mixture of CeO_2_ and CePO_4_ (Fig. 5B). This means that the interface between CePO_4_ and CeO_2_, formed during the hydrothermal treatment, is crucial for the strongly enhanced reactivity of the bifunctional nanocomposite during BA oxidation. It is important to note that the improved reactivity of the bifunctional nanocomposite may also result, to some extent, from a larger surface area of this material. As described in Sect. “Characterization of Catalysts”, the CeO_2_ particles in the bifunctional nanocomposite have a significantly smaller size than those in the sole CeO_2_. According to the literature^8^, concentration of defect sites in CeO_2_ increases with decreasing particle size. Thus, the smaller size of the ceria particles in the nanocomposite catalyst may result not only in a greater interface between CeO_2_ and CePO_4_, which facilitates redox switching between Ce^3+^  ↔ Ce^4+^ sites from the CePO_4_ and CeO_2_ lattice, but also in higher concentration of Ce^3+^ sites which play an important role in activation of ozone towards formation of hydroxyl radicals via reactions (1) and (2). Therefore, the strongly enhanced reactivity of the bifunctional CeO_2_/CePO_4_ system most likely results from the concentered effect of the enhanced activation of ozone activation by Ce^3+^  ↔ Ce^4+^ redox system and the elimination of the scavenging effect of chloride ions by the presence of surface phosphate groups.

## Conclusions

Results obtained in this study showed that solid cerium(III) phosphate enabled significantly higher ozonation recovery effect in the presence of chloride ions than homogeneous phosphates used in much greater amounts. It was also documented that the reactivity of the CePO_4_ can be further enhanced by preparation of a bifunctional nanocomposite containing both CeO_2_ and CePO_4_. Although the synergistic effect of CePO_4_ and CeO_2_ was clearly indicated by the results of this work, more detailed studies are necessary to fully understand the nature of synergy between the components of the bifunctional catalyst during ozonation processes and to unravel the mechanism of catalytic activation of ozone over CePO_4_/CeO_2_.

## Methods

### Chemicals and reagents

Cerium(III) nitrate hexahydrate (Ce(NO_3_)_3_ × 6H_2_O, Sigma-Aldrich, ACS Reagent, 99.99%), sodium hydroxide (NaOH, POCH, ACS reagent), ammonium phosphate dibasic ((NH_4_)_2_HPO_4_, Sigma-Aldrich, ACS reagent, ≥ 98%), potassium bromide (KBr, Sigma-Aldrich, FT-IR grade, ≥ 99%), hydrochloric acid (HCl, Chempur, 38%), perchloric acid (HClO_4_, Fisher Chemicals 60%) potassium indigotrisulfonate (Sigma Aldrich > 60%), *tert*-butyl alcohol (TBA, Sigma-Aldrich, ≥ 99.5%), benzoic acid (BA, Sigma-Aldrich ≥ 99.5%). All chemicals were used without further purification. Deionized water was used during synthesis. In ozonation and adsorption experiments high purity (Merck-Millipore) water was used.

### Synthesis of catalysts

In a typical synthesis route, 4.3422 g (0.01 mol) of cerium(III) nitrate hexahydrate was dissolved into 150 mL of deionized water. Then, 1.1999 g (0.03 mol) of sodium hydroxide dissolved in 50 mL of deionized water was stirred into the aqueous solution containing cerium(III) nitrate hexahydrate. Following 1 h of intensive agitation at room temperature, the resulting mixture was sealed into Teflon-lined stainless steel hydrothermal reactor and heated for 24 h at 100 °C. After cooling to room temperature, the solid formed during the hydrothermal treatment was then separated by centrifugation (8 000 RPM, 15 min), washed several times with deionized water and dried for 24 h at 80 °C. The as-prepared material was denoted as CeO_2_.

Cerium(III) phosphate (CePO_4_) and bifunctional CePO_4_/CeO_2_ nanocomposite were synthesized by the same hydrothermal procedure. For preparation of CePO_4_, 1.3206 g (0.01 mol) of ammonium phosphate dibasic ((NH_4_)_2_HPO_4_) was used instead of sodium hydroxide. In the case of the bifunctional CePO_4_/CeO_2_ catalyst, 1.1999 g (0.03 mol) of sodium hydroxide dissolved in 25 mL of deionized water was firstly stirred into the aqueous solution containing cerium(III) nitrate hexahydrate. Following 30 min of intensive agitation, 0.6603 g (0.005 mol) of (NH_4_)_2_HPO_4_ dissolved in 25 mL of deionized water was then added, and the resulting mixture was stirred for another 30 min at room temperature.

### Characterization of catalysts

The X-ray powder diffraction (XRD) measurements were carried out with a D8 Advance diffractometer (Bruker) using Cu Kα radiation (λ = 0.154 nm). The XRD patterns were acquired with a step size of 0.02◦ in the 2Θ range of 10–65°.

FTIR spectra of samples were acquired in the range from 4000 cm^−1^ to 400 cm^−1^ (resolution 4 cm^−1^, number of scans = 64) using a Bruker Vertex 70 spectrometer. For the FTIR measurements with KBr, the catalysts were dispersed in KBr pellet (2 mg of the sample and 200 mg of KBr).

The N_2_ adsorption–desorption isotherms were obtained at ‒196 °C using an ASAP 2020 Physisorption Analyzer (Micromeritics, USA). Before the measurements, the samples were degassed at 120 °C for 10 h. The specific surface area of the materials obtained was calculated by the Brunauer–Emmett–Teller (BET) method, and the average pore size was estimated by Barrett-Joyner-Halenda (BJH) method from the adsorption branch of the isotherm.

X-ray photoelectron spectroscopy (XPS) analyses were performed using an ultra-high vacuum photoelectron spectrometer based on Phoibos150 NAP analyzer (Specs, Germany). The analysis chamber was operated under vacuum with a residual pressure of 5 × 10^‒9^ mbar and the sample was irradiated with a monochromatic Al Kα (1486.6 eV) radiation. Any charging that would occur during the measurements was compensated for by shifting the entire spectrum by a distance needed to set the binding energy of the C 1 s, assigned to adventitious carbon, to the reference value of 284.8 eV.

TEM images of catalysts were recorded with the use of Hitachi HT7700 microscope (Hitachi, Japan) operating at 100 kV. Prior to the microscopic imaging, the samples were dispersed on nickel mesh covered with a carbon film. The CeO_2_ particle size distribution was calculated using ImageJ software^42^ by measuring the size of at least 200 particles.

### Catalytic tests

Catalytic tests were conducted in a semi batch mode at room temperature. Before each series of experiments, to satisfy the reaction vessel ozone demand, it was ozonated for 20 min. Next, the reaction vessel was filled up with ultrapure water acidified with 1 M HCl to pH 2.5 (total volume of acidified water = 200 mL). In experiments conducted without the chlorides, hydrochloric acid was replaced by the perchloric acid. The acidified water in the reaction vessel was then saturated by stream of ozone for 20 min. Initial and residual ozone concentrations in water was measured by the indigo method^43^. In all experiments, the average initial ozone concentrations were about 120 ± 5 µM. The reaction was started by addition of benzoic acid to the reaction vessel.

Quantification of benzoic acid (average starting amount 24 µM) in the samples withdrawn from the reaction vessel after given reaction time was done chromatographically, using a Symmetry C18 column (75 × 4.6 mm, 3.5 mm packing). Water/acetonitrile (50:50) was used as a mobile phase; water was acidified to pH 3.0 by H_3_PO_4_. The flow rate was set at 0.6 mL/min. Before the analyses, immediately after sampling, Na_2_SO_3_ (68 mM) was added to quench residual ozone. Each experiment was repeated at least three times. The RSD of measurements was lower than 3%. In order to investigate the role of hydroxyl radicals during ozonation process, additional experiments with the use of *tert*-butanol (4 mM) as HO^•^ scavenger were performed. In a typical experiment, 760 µL of *tert*-butanol (TBA : water solution volume ratio = 1 : 10) was added to the reaction vessel before the addition of benzoic acid.

Ozone decomposition experiments were performed at room temperature for 60 min. In each series of experiments the reaction vessel was fulfilled with ultrapure water acidified to pH 2.5 with the HClO_4_, and then saturated by ozone for 20 min. Immediately afterward zero sample was taken for O_3_ concentration measurements the reaction was started by addition of proper catalyst (40 mg).

The total organic carbon content in post-reaction mixtures was determined using the Total Organic Carbon analyzer (TOC-L) (Shimadzu, Japan).
